# Transcriptional cofactor Mask2 is required for YAP-induced cell growth and migration in bladder cancer cell: Erratum

**DOI:** 10.7150/jca.91041

**Published:** 2023-10-21

**Authors:** Liang Dong, Fan Lin, Wanjun Wu, Weiren Huang, Zhiming Cai

**Affiliations:** State Engineering Laboratory of Medical Key Technologies Application of Synthetic Biology, Shenzhen Second People's Hospital, The First Affiliated Hospital of Shenzhen University, Shenzhen 518039, PR China.

In the previously published version of this article, the representative image of the wound healing assay for 5637 cells overexpressing YAP in Figure 1G' is wrong. The updated Figure 1 is as follows. The correction made in this erratum does not affect the original conclusions. The authors apologize for any inconvenience or misunderstanding that this error may have caused.

## Figures and Tables

**Figure 1:**
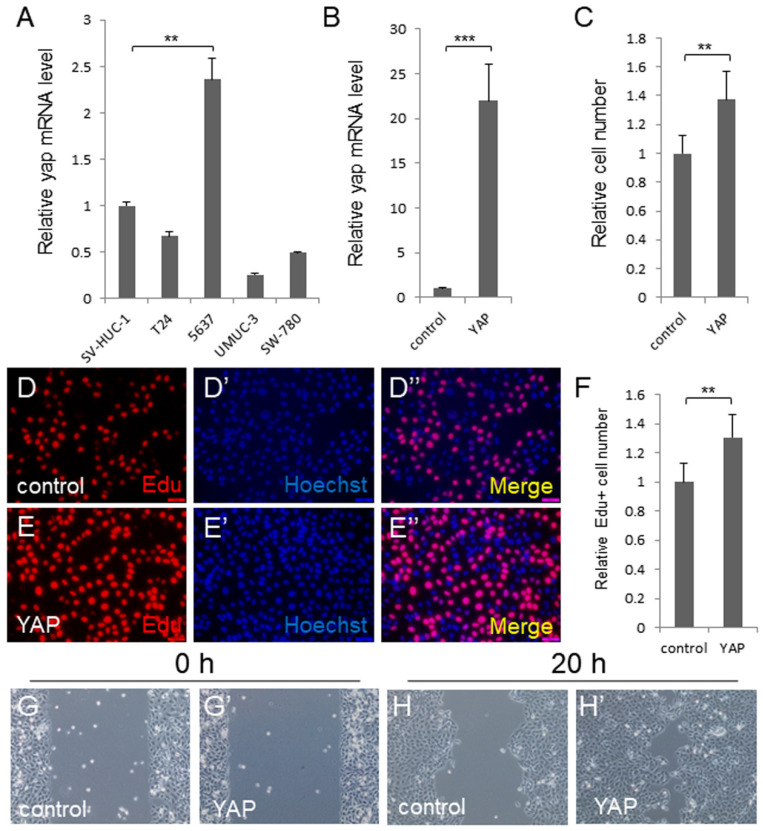
The corrected new figure is shown.

